# SDS, a structural disruption score for assessment of missense variant deleteriousness

**DOI:** 10.3389/fgene.2014.00082

**Published:** 2014-04-21

**Authors:** Thanawadee Preeprem, Greg Gibson

**Affiliations:** School of Biology, Georgia Institute of TechnologyAtlanta, GA, USA

**Keywords:** non-synonymous single nucleotide polymorphism, missense mutation, protein structural analysis, structural disruption score, variant prioritization, epilepsy disorders

## Abstract

We have developed a novel structure-based evaluation for missense variants that explicitly models protein structure and amino acid properties to predict the likelihood that a variant disrupts protein function. A structural disruption score (SDS) is introduced as a measure to depict the likelihood that a case variant is functional. The score is constructed using characteristics that distinguish between causal and neutral variants within a group of proteins. The SDS score is correlated with standard sequence-based deleteriousness, but shows promise for improving discrimination between neutral and causal variants at less conserved sites. The prediction was performed on 3-dimentional structures of 57 gene products whose homozygous SNPs were identified as case-exclusive variants in an exome sequencing study of epilepsy disorders. We contrasted the candidate epilepsy variants with scores for likely benign variants found in the EVS database, and for positive control variants in the same genes that are suspected to promote a range of diseases. To derive a characteristic profile of damaging SNPs, we transformed continuous scores into categorical variables based on the score distribution of each measurement, collected from all possible SNPs in this protein set, where extreme measures were assumed to be deleterious. A second epilepsy dataset was used to replicate the findings. Causal variants tend to receive higher sequence-based deleterious scores, induce larger physico-chemical changes between amino acid pairs, locate in protein domains, buried sites or on conserved protein surface clusters, and cause protein destabilization, relative to negative controls. These measures were agglomerated for each variant. A list of nine high-priority putative functional variants for epilepsy was generated. Our newly developed SDS protocol facilitates SNP prioritization for experimental validation.

## Introduction

Several prediction programs are available to evaluate missense variants as either deleterious (having a strong functional effect) or neutral (having no or only a weak functional effect) from the level of DNA or protein sequence conservation (Cooper and Shendure, 2011). While existing sequence-based damaging scores agree for the most deleterious variants, predictions for candidate moderate effect variants identified from sequencing studies are not much better than chance. Since there is no clear way to truly evaluate the predictive accuracy of the scores prior to experimental assessment of function, there is scope for development of orthogonal methods for variant prioritization. Our study explores the utility of solely using protein structure-based assessments as a complement to existing sequence-based scores.

Of the commonly used automatic tools for prediction of variant deleteriousness, PolyPhen2 (Adzhubei et al., 2010) already incorporates protein structure information. It uses an iterative greedy algorithm to select certain features from a restricted training set, and then takes a Bayesian approach to assign each variant into one of four effect categories: probably damaging, possibly damaging, benign, and unknown. However, it does not perform evaluations on the actual protein structure that each variant is found in. Rather, PolyPhen2 includes experimentally derived-structures that are available for ~10% of the training set. Although the implementation has high accuracy (73–92%) for the identification of true positives in cross-validation data, structural data does not directly contribute to evaluations of novel genes and it is not clear how efficiently the generalized structural characteristic rules used by the algorithm can contrast clinically-associated variants from neutral variants in a diverse gene set.

In this study, we therefore introduce a new approach for assessing the deleteriousness of non-synonymous single nucleotide polymorphisms (nsSNPs). Our newly developed protocol uses additional information, that is, protein structure-based assessments applied only where the structural solution is available, to complement existing sequence-based scores. More specifically, our evaluation pipeline focuses on functionality of protein residues derived from 3-dimensional (3D) protein structures. We also incorporate multiple classes of structural assessment, namely measures of protein stability, flexibility, protein-protein interaction potential, and small-molecular binding. As several studies (Capriotti and Altman, 2011; Jordan et al., 2011) have proven that structural information increases classification accuracy of SNPs, we hypothesized that by incorporating results from several structure-based assessments, it may be possible to generate characteristic profiles that enhance prediction of the degree to which a candidate rare variant may disrupt protein function, and lead to disease development.

We applied this newly developed variant assessment protocol to a set of 57 gene products harboring homozygous missense variants, discovered in a recent large-scale exome sequencing study, that are exclusive to epilepsy patients (Heinzen et al., 2012). Epilepsy is a highly genetically heterogeneous disease, for which each likely causal variant is observed in a small fraction of individuals, likely with variable expressivity and penetrance (Noebels, 2003). As a result, it is difficult to ascertain which variants are truly responsible for the etiology of disease in individual patients. None of the case-exclusive variants documented by Heinzen et al. (2012) had a high enough prevalence to support statistical association with the disease, so experimental tests will be needed to filter putative causal variants. By contrasting the spectrum of structural features of the case variants with positive control known causal variants and negative control neutral variants observed in healthy individuals for the same proteins, we illustrate the potential for structural assessment to prioritize new variants for functionalization.

## Materials and methods

**Figure 1:**
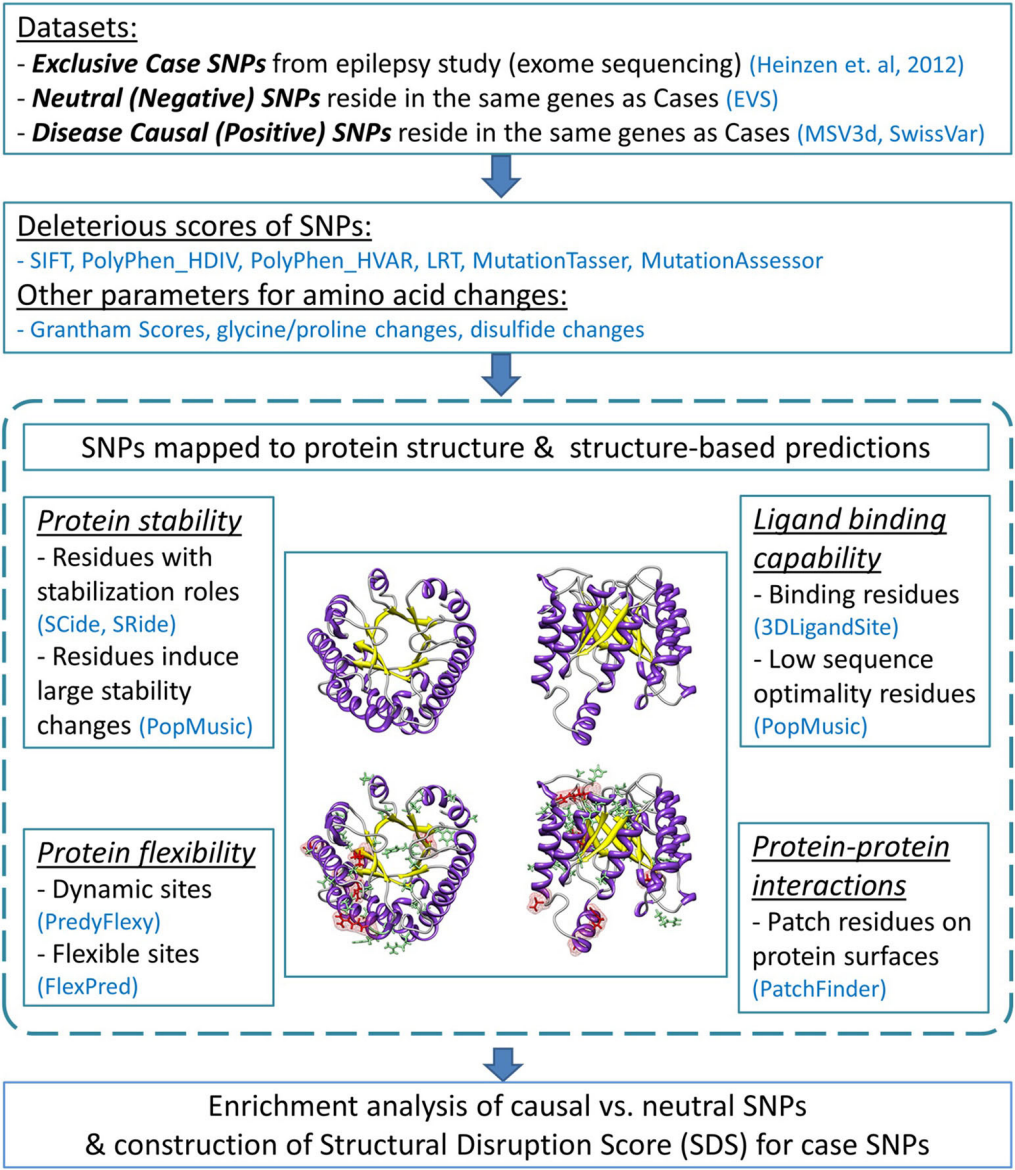
**Flow diagram of the analysis pipeline**. The analysis employed sequence-based deleterious prediction scores, parameters which reflect the nature of amino acid changes, and 3D structure-based evaluations. Structural analyses were performed by characterizing functionality of mutated protein residues caused by negative and positive SNPs (indicated by green and red stick representations, respectively). All analysis results were collectively used to evaluate enriched features found predominantly in causal SNPs. We then examined these predictors with regard to the case variants. The number of deleterious structure predictions per substitution represents a “structural disruption score” (SDS), and was used to rank candidate epilepsy variants.

Our analysis pipeline applied sequence- and structure-based assessments to missense mutations and their 3D protein structures to depict the likelihood that a mutation disrupts protein function. Numerous databases and prediction programs were used. The flow diagram of the analysis protocol is illustrated in Figure 1.

### Genomic dataset and candidate protein sequences

The epilepsy-specific amino acid substitutions identified from a recent exome sequencing study of epilepsy disorders (Heinzen et al., 2012) served as our case variants for which we aimed to assess whether or not they are likely to impact protein function. In that study, exome sequencing was performed on 118 cases and 242 controls. Follow-up genotyping for candidate causal variants included approximately 90 and 65% of individuals with European ancestry in the case (*n* = 878) and control (*n* = 1830) groups, respectively (Heinzen et al., 2012). The study identified 72 homozygous variants (68 are nsSNPs) found in 71 genes (“**gene set 1**”) that were exclusive to cases. Among these, 52 nsSNPs were present in more than one affected individual. All genes in this first dataset had been previously characterized but not known to cause epilepsy; therefore, we added a second gene set (“**gene set 2**”) to represent genes known to associate with the disorders. We attained the second gene list (*n* = 41 genes) from two public repositories of genetic variations: MSV3d (Luu et al., 2012) and SwissVar (Mottaz et al., 2010); none of the genes overlap with entries from the primary dataset. There are 373 missense variants in the 41 genes that have been documented to cause epilepsy; therefore, we treated them as case variants for gene set 2.

For both sets of genes, we compiled corresponding negative neutral and positive causal variants from the EVS database (retrieved March 2013) (NHLBI Exome Sequencing Project, 2013), and MSV3d (July 2012 release) (Luu et al., 2012) and SwissVar (accessed February 2013) (Mottaz et al., 2010), respectively. Positive controls are documented non-epileptic disease-causing nsSNPs found in the same genes (*n* = 134 nsSNPs from 14 genes of set 1, and *n* = 205 nsSNPs from 41 genes of set 2). Likewise, negative controls are variants observed in these genes, but with no clinical associations (neutral nsSNPs). Any negative controls already identified as either case or positive SNPs were excluded from the list of neutral SNPs, resulting in 5281 and 1490 putatively neutral (i.e., negative control) SNPs for sets 1 and 2, respectively.

### Gene and variant annotations

In order to infer amino acid indices for the altered amino acid residues, nsSNPs were mapped to their corresponding protein sequences and structures using transcript IDs. All protein sequences (major isoforms) were downloaded from the UniProt database (accessed February 2013) (Uniprot Consortium, 2012). Prior to applying our new variant analysis protocol, we performed literature searches on the genes and SNPs in our datasets in order to manually annotate their influence on the disease. In particular, we compared the features of gene sets 1 and 2, and recorded relevant findings.

First we grouped genes by their related biological pathways or biological functions using a gene group profiling method (Reimand et al., 2011). Second, we performed literature searches using SNPshot—a text mining tool for PubMed abstracts (accessed December 2012) (Hakenberg et al., 2012). Third, we assumed that amino acid mutations caused by the rare case SNPs or the causal SNPs would locate in the vicinity of functional sites of protein chains. Therefore, we utilized UniProt's sequence feature records (accessed February 2013) (Uniprot Consortium, 2012) to check if the mutating amino acids locate in any of the important sites, e.g., molecule processing sites, binding sites, modification sites, etc.

Population-specific minor allele frequencies (MAFs) for all variants were compiled from NHLBI GO Exome Sequencing Project (ESP6500) (June 2012 release), available from dbNSFP 2.0 (accessed March 2013) (Liu et al., 2011).

### Protein structure dataset

We used protein 3D structures to determine the structural nature of altered protein residues and to evaluate the effects of single point mutations introduced by nsSNPs on a specific protein. To ensure that we represent most of the proteins with high quality 3D structures, we employed several structural sources. Experimentally derived structures were retrieved from RCSB (retrieved April 2013) (Bernstein et al., 1977). Homology models were compiled from SAHG (retrieved July 2013) (Motono et al., 2011) or automatically built using Phyre2 (accessed April 2013) (Kelley and Sternberg, 2009). Multiple structural candidates representing an overlapping protein chain were compared and only one best structure was chosen to represent the best non-overlapping protein segment.

Details of the two approaches for acquiring protein homology models are as follows. First, we searched for 3D models from the SAHG database (Motono et al., 2011), which contains a collection of encoded human protein structures, constructed by Modeller software (Sali and Blundell, 1993). We downloaded only structures having >15% sequence identity to the template. The retrieved proteins exhibit either ligand bound (holo) and/or unbound (apo) forms. Second, we built protein models by multiple template methodology using the automated Phyre2 homology modeling server (Kelley and Sternberg, 2009). Structure templates were selected by default and models were built from variable numbers of high confidence templates. This multi-template approach ensures that the model covers most of the protein chain. Large proteins (>1200 amino acids) were truncated into smaller domain(s) using domain boundary information from Interpro (Quevillon et al., 2005). A model representing each shortened sequence was built independently using either the single- or multi-template method; there was no attempt to join multiple models into a single model for a protein. For models created with the Phyre2 server, we retained the best homology model based on the empirical criteria that >50% of the residues were modeled at >90% confidence.

After the initial homology model selection, the models were further subjected to energy minimization with explicit solvent using the YASARA force field (Krieger et al., 2009) to resolve any steric conflicts found within the structures. Next, we validated the homology models using two independent scores: QMEAN6 (Benkert et al., 2009) and ModFOLD4 (McGuffin et al., 2013). Both scores show good ability to distinguish between good and bad models in the recent Critical Assessment of protein Structure Prediction **(**CASP) experiments (Kryshtafovych et al., 2014). To facilitate the structural validation step, we selected structures that pass the QMEAN6 threshold for subsequent ModFOLD4 evaluations.

In many cases, we initially selected more than one validated structure to represent an identical protein domain. To retain only one best representative structure for a protein segment, we used Chimera (Pettersen et al., 2004) to visualize all structure candidates and determined the structural similarity among them using two parameters: root-mean-square deviation (RMSD) of C_α_ atoms, and quality score (Q-score) that normalizes an RMSD by the alignment length. All measurements were performed with Chimera's MatchMaker tool (Pettersen et al., 2004). When several overlapping structures agreed with each other, we selected the one with the best ModFOLD4 score. When the structures were in disagreement, we discarded them all together. Our retrieval and validation pipeline for protein 3D structures yielded 114 non-overlapping structures representing 57 gene products from gene set 1, and 51 non-overlapping structures representing 36 proteins from gene set 2.

Table 1 summarizes the number of missense variants from our genomic dataset in three categories (case, negative, and positive controls), with respect to the presence/absence of their corresponding 3D structures.

**Table 1:** **Number of variants within each gene set, classified into three classes (cases, negative controls, and positive controls), and numbers of 3D structures used in the analysis**.

**Gene set**	Number of variants by categories^*^			Number of 3D structures by types and sources^**^					**Total # of selected structures**	**# of genes with selected structures**
	**Case**	**Neg**	**Pos**	**Crystal structures**	**Homology models**					
				**RCSB**	**Phyre2 (multi-template)**	**Phyre2 (single-template)**	**SAHG (apo)**	**SAHG (holo)**		
Set 1 (71 genes)	30 (68)	1674 (5281)	100 (134)	20 (24)	8 (35)	35 (59)	20 (86)	31 (80)	114	57
Set 2 (42 genes)	184 (373)	554 (1490)	105 (205)	2 (2)	3 (19)	5 (17)	21 (46)	20 (38)	51	36

* *Number of variants by categories is indicated by the number of SNPs locate within the set of selected 3D structures (114 structures for gene set 1, and 51 structures for gene set 2), followed by the total number of SNPs with and without 3D structures (number shown in parentheses)*.

** *Number of structures represents the number of selected 3D structures that passed quality validation scores. The initial number of structures obtained from each data source is much larger, indicated by numbers in parentheses*.

### Inferring variant deleteriousness from sequence-based predictors

We obtained sequence-based predictions for each amino acid variant from dbNSFP 2.0 (accessed March 2013) (Liu et al., 2011). The program provides precomputed deleteriousness scores for six established deleterious prediction algorithms: SIFT (Kumar et al., 2009), PolyPhen2_HumDiv and PolyPhen2_HumVar (Adzhubei et al., 2010), LRT (Chun and Fay, 2009), MutationTaster (Schwarz et al., 2010), and MutationAssessor (Reva et al., 2011). Three evolutionary conservation-based scores were also included: GERP++ (Davydov et al., 2010), phyloP (Pollard et al., 2010), and SiPhy (Lindblad-Toh et al., 2011). For simplicity, we assigned the level of deleteriousness and conservation to each mutation based on how many predictors reported the mutation to be either “deleterious” (maximum score of 6) or “conserved” (maximum score of 3).

### Additional parameters for sequence-based analysis

In addition to the SNP-based prediction parameters that are derived from multiple sequence alignments, some useful information can be analyzed from a single protein sequence alone. For example, amino acids with similar physicochemical properties may substitute for one another while maintaining the functionality of the protein. Three indicators may be used to highlight the most severe changes of amino acid pairs. First, Grantham scores (Grantham, 1974) reflect the degree of physico-chemical difference between pairs of amino acids. Second, changes involving any glycine or proline residues are likely to affect protein function since these two residues have special roles with regard to protein structure: proline has an exceptional conformational rigidity compared to other amino acids while glycine is much more conformationally flexible (Gunasekaran et al., 1998). Third, gain or loss of disulfide bonds occurs when variants induce changes in cysteine residues. Disulfide bond formation between non-adjacent cysteines can facilitate protein folding; hence, they are important for maintaining the structural integrity of the protein (Darby and Creighton, 1995). In this context, we used DiANNA webserver (Ferre and Clote, 2006) to predict the disulfide connectivity patterns in the wild type protein, and then determined if the amino acid mutation affects the bonding of cysteine pairs.

### Inferring variant deleteriousness from structure-based predictors

A number of currently available protein structural analysis tools have the potential to be applied to structure-based variant assessment protocols (Verma et al., 2012). To assess the functionality of mutated protein residues, we concentrated on four features of structural analysis: protein stability, protein flexibility, protein-ligand binding potential, and protein-protein interaction potential. Many mutations disrupt these elements, and as a result, contribute to disease etiology.

#### Protein stability

For assessment of protein stability, we aimed to first identify amino acids with specialized roles in promoting protein stability, and second to determine which mutations cause a significant change in protein stability. For the first objective, we used SCide webserver (Dosztanyi et al., 1997, 2003) and SRide program (Magyar et al., 2005) to identify amino acids with essential stability functions. Long-range stabilization center (SC) residues are pairs of amino acids having close atomic contact (sum of van der Waals radii <1 Å), but locate at least ten amino acids apart on the primary sequence (Dosztanyi et al., 1997, 2003). A subset of SC residues may make distinct contributions to protein stability because they are also evolutionary conserved and located in the core region of the protein, and/or have many interacting partners. SC residues with these two extra properties are referred as stabilizing residues (SRs); they are also expected to make key contributions to protein stability (Magyar et al., 2005). For the second objective, we aimed to determine if a particular mutation affects protein stability by means of inducing a large magnitude of free energy change (ΔΔG). For this purpose, we selected PoPMusic 2.1 (Dehouck et al., 2011) as our ΔΔG predictor.

Amino acid changes that increase protein stability (ΔΔG < 0) and those associated with the destabilizing mutation (ΔΔG > 0) are noted. Due to large differences in performance of stability change calculations (Khan and Vihinen, 2010), the proper margin for severe stability change can be ambiguous. However, it is known that the sensitivity in predicting stabilizing mutations is much less than for destabilizing ones (Worth et al., 2011), and the correlation between predicted stability change (ΔΔG_P_) and measured values (ΔΔG_M_) of our selected program is ~1 kcal/mol (Dehouck et al., 2011). Therefore, in our study, we followed the suggestions made by Dehouck et al. (2011). The stability changes are categorized into four levels: no change if ΔΔG is between ±0.5 kcal/mol, mildly stabilizing if ΔΔG is between −0.5 and −2 kcal/mol, mildly destabilizing if ΔΔG is between 0.5 and 4 kcal/mol, and strongly destabilizing if ΔΔG is ≥4 kcal/mol.

#### Protein flexibility

Protein flexibility is an important protein feature because highly dynamic sites are often involved in special functions, such as binding residues that can undergo subtle motion rearrangements when a small molecule is bound. Flexible amino acid residues permit large protein movements during protein folding and conformational switches (Teilum et al., 2009). For evaluating the levels of residue dynamics within a protein, we employed the predicted B-factors (relative vibrational motion) and root-mean-square fluctuations (RMSFs) obtained from a prediction program PredyFlexy (de Brevern et al., 2012) to classify amino acid residues into rigid, intermediate, or flexible sites. For predicting protein movements of higher amplitudes, such as in conformational switches, we used the program FlexPred (Kuznetsov, 2008; Kuznetsov and McDuffie, 2008) to determine which amino acid residues are located at conformationally flexible sites, indicated by a probability value P(Flexible).

#### Protein-ligand binding potential

For a SNP that causes an amino acid change in the vicinity of a catalytic site or a ligand binding site, it is possible to determine whether the mutation is indeed affecting the catalytic activity or the ligand binding affinity of the protein. *In silico* predictions are possible, but they require extensive computational resources. We utilized two alternative approaches to predict the ligand binding sites or catalytic sites from protein 3D structures, and assessed whether or not the altered protein residues locate in or near the predictions. The first approach began with the use of 3DLigandSite (Wass et al., 2010) to search for ligands present in homologous structures. Then, a cluster of amino acids located within a default distance setting of 0.8 Å of the selected ligand was predicted as a pocket site, and amino acid residues that make up that pocket site were specified as ligand binding residues. In the second method, catalytic sites were predicted by scanning for protein residues that are not well optimized. This assumption is based on the finding that catalytic sites are generally designed for function rather than stability (Dehouck et al., 2011). Low optimality residues are those whose several possible mutations would improve protein stability. The program PoPMusic (Dehouck et al., 2011) is fast enough that it can calculate stability changes (ΔΔGs) of all possible mutations at a given position in the protein sequence, and was used to identify non-optimized amino acid residues based on the summation of all stabilizing ΔΔGs. This parameter designates the degree of non-optimality (Γ) for each amino acid residue along the protein chain.

#### Protein-protein interaction potential

Disease-causing mutations that do not occur in binding sites or buried sites are predominantly found on protein interfaces (David et al., 2012); therefore, we assessed which of the mutating protein residues may be involved in this type of inter-molecular interaction. We used PatchFinder program (Nimrod et al., 2005, 2008), which evaluates evolutionary conservation scores in conjunction with solvent accessibility of protein residues, to determine the most significant cluster of conserved residues on the surface of a protein. This patch indicates possible functional sites of protein-protein interactions.

### Additional parameters for structure-based analysis

Other information retrieved from the structure-based data includes the type of protein secondary structure that each variant interrupts, and the relative solvent accessibility (RSA) of the altered protein residue. We obtained these predictions from PoPMusic (Dehouck et al., 2011). Due to the small sample size, we modified the eight reported types of protein secondary structure (Kabsch and Sander, 1983) into five groups: (G/H/I), E, (T/B), S, and C. The 3-, 4-, and 5-turn helices (groups G, H, and I, respectively) were grouped jointly as helices. Extended strand in parallel and/or anti-parallel β-sheet remains as an individual group (group E). Groups T or B correspond to helices or sheets whose hydrogen bonding patterns are too short to form proper secondary structures. Lastly, groups S and C denote bend and coil annotations, respectively.

The RSA for residue X is expressed as a percentage of that observed for an Alanine-X-Alanine tripeptide. This conformation would expose the central X residue in the tripeptide as much as would normally be possible in a protein (Dehouck et al., 2011). We considered protein residues whose RSA ≤ 20% as buried sites. Otherwise, they are expected to be on the protein surface.

### Statistical comparison of positive and negative SNPs

We tested which predictions and measures can statistically distinguish between negative and positive control SNPs from each gene group. Particularly, we assessed which characteristics are most likely to be enriched in positive controls, and therefore imply disruption of functionality. After defining thresholds of likely deleterious function, we classified the predicted values for negative and positive controls into each different category. The categories for structural indicators, the numerical cutoff values, and the numbers of mutations with extreme values from the two datasets are summarized in Table 2.

**Table 2:** **Categories for structural indicators, cutoff values for continuous numerical parameters, and number of SNPs with extreme measures**.

**Indicators**	**Cutoff**	**# Case (%) (*n* = 30)**	**# Neg (%) (*n* = 1674)**	**# Pos (%) (*n* = 100)**
Stability change	*Stabilizing*: ΔΔG between −2 and −0.5 kcal/mol	0	19 (1%)	0
	*Strong stabilizing*: ΔΔG ≤ −2 kcal/mol	0	0	0
	*Destabilizing*: ΔΔG between 0.5 and 4 kcal/mol	20 (65%)	830 (50%)	66 (66%)
	*Strong destabilizing*: ΔΔG ≥ 4 kcal/mol	0	1 (0%)	0
Dynamic sites	*Highly rigid*: B-factor_norm_ ≤ −0.537 (@2.5%)	0	38 (2%)	3 (3%)
	*Highly dynamics*: B-factor_norm_ ≥ 1.17 (@97.5%)	1 (3%)	44 (3%)	1 (1%)
Dynamic sites	*Highly rigid*: RMSF_norm_ ≤ −0.607 (@0.5%)	0	48 (3%)	2 (2%)
	*Highly dynamics*: RMSF_norm_ ≥ 1.195 (@99.5%)	1 (3%)	48 (3%)	3 (3%)
Flexible sites	*Conformationally rigid*: P(Flexible) ≤ 0.158 (@2.5%)	1 (3%)	34 (2%)	2 (2%)
	*Conformationally flexible*: P(Flexible) ≥ 0.860 (@97.5%)	1 (3%)	52 (3%)	0
Sequence optimality	*Highly non-optimal*: Γ ≤ −5 kcal/mol	1 (3%)	49 (3%)	5 (5%)

*All cutoff values were defined exclusively for gene set 1. The counts and percentages of variants with extreme values in each of the three variant classes are included*.

**Figure 2:**
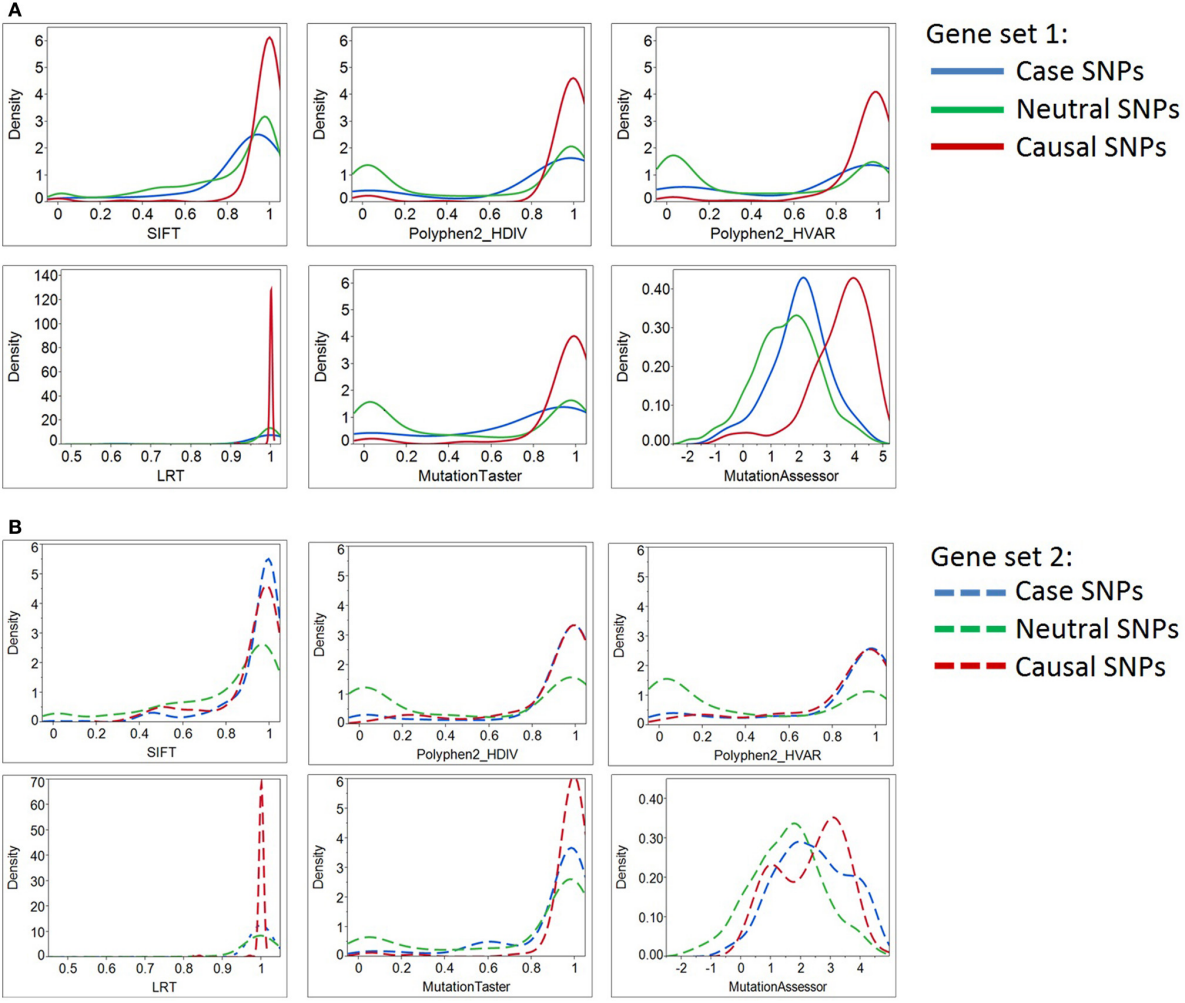
**Density plots of six deleterious scores for Case, Neutral, and Causal SNPs**. By most of the standard deleteriousness scores, the distributions of cases in gene set 1 (Panel **A**) are closer to the neutral than the causal variants, and the neutral and causal variants are significantly different. The “known epilepsy” dataset (gene set 2, Panel **B**) demonstrated similar results. In this gene set, variants documented to cause epilepsy were regarded as “cases,” while variants associated with other disease types were considered as “causal SNPs (positive control).” Although three prediction programs (SIFT, Polyphen2_HDIV, and Polyphen2_HVAR) suggested case and causal SNPs share similar distributions of deleterious scores, the remaining three programs illustrate their prediction algorithms do not favor the two types of causal variants equally. More importantly, case SNPs (epilepsy-causing SNPs) resemble neutral SNPs more than the causal ones.

**Figure 3:**
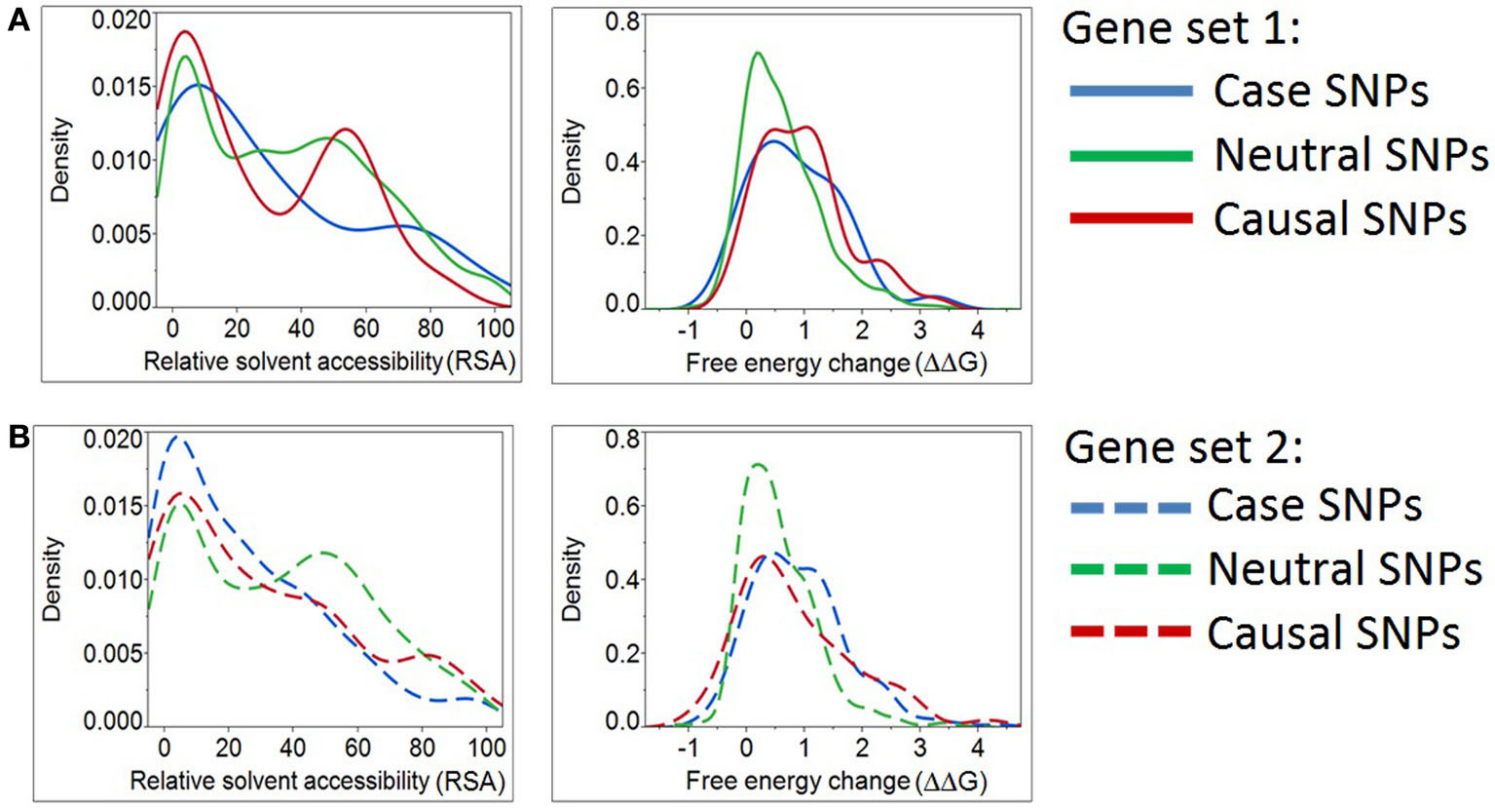
**Density plots for relative solvent accessibility and free energy change for Case, Neutral, and Causal SNPs**. The two structure-based scores demonstrate that Case and Causal SNPs share similar characteristics. Results were obtained from two independent sets of genes (Panels **A,B**). Note the shift of the blue curves (cases) toward the causal SNPs (red) and away from the neutral ones (green).

For continuous parameters, we compared the difference between the means of the positive and negative controls using two-tailed unpaired *t*-tests (Table 3). The distributions of scores within each SNP group were also illustrated by density plots (Figures 2, 3). For non-numerical characteristics, we used Fisher's exact test to determine whether the proportions of negative and positive control SNPs for each of the features are significantly different (Table 4). For continuous measures, similar analyses were performed after first transforming the scores into discrete categories based on prespecified thresholds that are most likely to discriminate normal and aberrant residues. Once a series of predictions and measures was generated for all possible variants in a gene set, we converted continuous parameters into categorical classes, utilizing both literature-based and empirical cutoff values to represent the extremes (Table 2).

**Table 3:** ***T*-test statistics for gene sets 1 and 2**.

**Parameters**		**Prob > |t|, (t ratio), df^†^**					
		**Set 1 (57 genes)**			**Set 2 (36 genes)**		
Sequence-based deleterious scores	SIFT	<0.0001^*^	(6.60)	df 1704	<0.0001^*^	(4.61)	df 598
	PolyPhen2_HDIV	<0.0001^*^	(8.15)	df 1772	<0.0001^*^	(7.38)	df 657
	PolyPhen2_HVAR	<0.0001^*^	(10.22)	df 1772	<0.0001^*^	(8.70)	df 657
	LRT	<0.0001^*^	(4.19)	df 1734	0.0066^*^	(2.73)	df 654
	MutationTaster	<0.0001^*^	(9.39)	df 1674	<0.0001^*^	(5.57)	df 631
	MutationAssesssor	<0.0001^*^	(15.30)	df 1772	<0.0001^*^	(6.06)	df 652
Sequence conservation scores	GERP	<0.0001^*^	(5.30)	df 1772	<0.0001^*^	(3.92)	df 657
	phyloP	<0.0001^*^	(4.81)	df 1772	0.0010^*^	(3.29)	df 657
	SiPhy	<0.0001^*^	(5.89)	df 1771	<0.0001^*^	(4.95)	df 657
Structure-based scores	ΔΔG	<0.0001^*^	(4.81)	df 1772	<0.0001^*^	(3.83)	df 657
	B-factor	0.0190^*^	(−2.35)	df 1756	0.2450	(1.16)	df 657
	RMSF	0.2304	(−1.20)	df 1756	0.2264	(1.21)	df 657
	P(Flexible)	0.1185	(−1.56)	df 1772	0.7594	(−0.31)	df 657
	Γ	0.9090	(−0.11)	df 1772	0.2308	(−1.20)	df 657
	RSA	0.0035^*^	(−2.93)	df 1772	0.0658	(−1.84)	df 657

*All tests were performed on a subset of SNPs whose protein structures pass quality validations. Significant statistics indicate different means between negative and positive SNPs*.

† Statistic parameters include the two-tailed p-value, value of the t-statistics (t ratio), and the degree of freedom (df). Significant p-values (α = 0.05) are designated with “

* *.” The number of “df” equals to n-2 samples used in the analysis*.

**Table 4:** **Fisher's exact test statistics for gene sets 1 and 2**.

**Enrichment types**	**Significant features**	**Fisher's exact test (one-tailed)**	
		**Set 1 (57 genes)^†^**	**Set 2 (36 genes)^‡^**
Enriched in causal SNPs	Deleterious count ≥ 4	<0.0001^*^	<0.0001^*^
	Conservation count = 3	Enriched in neutral SNPs	<0.0001^*^
	Induce large amino acid change (Grantham score ≥ 100)	<0.0001^*^	<0.0001^*^
	Induce disulfide change	NS (*p* = 0.6081)	<0.0001^*^
	Induce glycine/proline change	0.0003^*^	<0.0001^*^
	Locates in buried site (RSA ≤ 20%)	0.0109^*^	<0.0001^*^
	Locate in conformationally rigid site (P(Flexible) ≤ 0.74)	NS (*p* = 0.0558)	0.0060^*^
	Locate on protein patch	<0.0001^*^	<0.0001^*^
	Locate in protein domain	0.0204^*^	<0.0001^*^
	Strongly reduce protein stability (ΔΔG ≥ 4 kcal/mol)	NS (*p* = 1)	0.0400^*^
	Reduce protein stability (ΔΔG ≥ 0.5 kcal/mol)	0.0009^*^	<0.0001^*^
Enriched in neutral SNPs	Conservation count = 3	<0.0001^*^	Enriched in causal SNPs
	Locate in highly dynamics site (B-factor_norm_ ≥ 97.5%)	NS (*p* = 0.2671)	0.0224^*^
	Locate in highly flexible site (P(Flexible) ≥ 97.5%)	0.0468^*^	NS (*p* = 0.1432)

High sequence conservation (conservation count = 3) is a feature that enriched in causal SNPs of gene set 2, but is more likely to be found in neutral SNPs of gene set 1.

† Data for gene set 1 includes 100 causal SNPs and 1674 neutral SNPs.

‡ Data for gene set 2 includes 289 causal SNPs (184 epilepsy case variants plus 105 non-epilepsy positive control variants), and 554 neutral SNPs.

Significant p-values (α = 0.05) are designated with “

* *.” Non-significant test statistics are labeled with NS, followed by the correspondent p-value*.

For protein stability and sequence optimality measures, we followed the suggested thresholds from the PoPMusic program (Dehouck et al., 2011). Stability changes were classified into four groups (mildly stabilizing, strongly stabilizing, mildly destabilizing, and strongly destabilizing) using the aforementioned cutoffs for ΔΔG. Regarding sequence optimality, it has been shown that as the threshold for Γ becomes more stringent, the proportion of catalytic sites to other sites increases (Dehouck et al., 2011). For our analysis, we selected a cutoff for which residues having Γ ≤ −5 kcal/mol are more likely to locate in ligand-binding domains.

For the remaining continuous measures, i.e., B-factor, RMSF, and P(Flexibility), we used empirical criteria to define the extremes. Extreme values for data set 1 and 2 were derived independently, but with an identical approach. First, we compared the score distributions of each measurement, collected from all possible SNPs in the protein set. Then, we selected the thresholds for each parameter so that we captured a handful of extreme variants. When applicable, we made sure that our thresholds do not induce large numbers of misclassifications. In summary, our empirically-defined thresholds are generally set at the top and bottom 2.5 percentiles. B-factor and RMSF predictions were classified into either highly rigid (extreme small negative values) or highly dynamic (extreme large positive values). Similarly, we denoted residues as conformationally rigid if the P(Flexibility) is exceptionally low, or conformationally flexible if the probability is notably high.

### Assigning a structural disruption score to candidate epilepsy variants

After testing for statistically significant differences between negative and positive SNPs, we summarized the list of deleterious structure predictions and examined these predictors with regard to the case variants. We counted the number of deleterious structure predictions per substitution and represented this number as a “structural disruption score” (SDS). The scores were ranked and candidate epilepsy variants with a score of ≥ 4 out of 7 are suggested to be “putative structural disrupted variants.” Further partitioning of this list based on the gene's tolerance of polymorphism, RVIS (Petrovski et al., 2013), yields two subgroups: variants of high tolerance genes (genes that have more variants than expected), and variants of low tolerance genes (genes that have less variants than expected). These two groups of variants are also discussed in detail with respect to their disease implications.

To examine the contribution of each selected parameter, especially the sequence-based deleterious score, toward our SDS, we compared the values of SDS with the Condel composite score (Gonzalez-Perez and Lopez-Bigas, 2011), derived from three of the deleteriousness measures (SIFT, PolyPhen, and MutationAssessor). The evaluation was performed with a step-wise procedure. First, we tested for a correlation between the Condel score and SDS—including all parameters from the sequence-based and structure-based predictions (total *n* parameters). Then, we re-evaluated the correlation using *n-1* parameters, by excluding one of the SDS components at a time.

## Results

### Candidate gene and variant annotations

Despite the fact that Heinzen et al. (2012) performed pathway analysis on 1183 genes harboring either homo- and/or heterozygous nsSNPs, with a genotype exclusive to the case group, no significantly over-represented biological pathways were found (Heinzen et al., 2012). Using an alternative gene group profiling method (Reimand et al., 2011), we also did not observe a statistical abundance for any biological terms derived from gene set 1. However, this method did reveal that ~40% of genes in set 2 have roles in transmission of nerve impulses, ion channel complexes, or ion gated channel activity. A text mining method (Hakenberg et al., 2012) discovered only 1 gene from set 1 that may be linked to epilepsy. This gene codes for a ubiquitin-like modifier activating enzyme 2 (UBA2), a drug metabolizing enzyme that plays roles in GABAergic and cholinergic neuronal development (Kitamura et al., 2009). Specifically, mutations in ubiquitin protein ligase along with disruptions in the important neuronal GABA receptor genes are suggested to induce seizure (Olsen, 2011). By contrast, all genes in set 2 are suspected to be involved in a wide range of epilepsy disorders (Mottaz et al., 2010; Luu et al., 2012). Also note that the proportion of variants in cases relative to controls is much lower for genes in set 1 than in set 2 (Table 1).

Annotation of altered amino acid residues indicated some consistent patterns between case and causal variants. When we performed sequence feature searches (Uniprot Consortium, 2012) to compute the number of variants localizing in structurally or functionally important sites of a protein chain, we observed that more than half of the positive SNPs in both gene sets 1 and 2 were predicted to locate in transmembrane, topological domain, or repeat regions. Similar patterns were found for the case-exclusive epilepsy variants.

### Statistically significant differences between positive and negative SNPs

Table 3 documents that all deleteriousness scores, all conservation scores, and some of structure-based scores have significantly different means between negative and positive SNPs. The single parameters that best differentiate the groups are the MutationAssesssor prediction for gene set 1 and the PolyPhen2_HVAR prediction for gene set 2, but more notable is the highly significant differentiation for all of the sequence-based scores. Although the t-ratios are consistently lower in set 2 than set 1, the smaller sample of genes precludes inference that there is a difference between the two sets. Notably, three of the structure-based measures are also at least nominally significantly different between positive and negative control variants in set 1, and trend in the same direction in set 2: ΔΔG protein stability, B-factor protein flexibility, and RSA.

We converted several of the continuous structural measures to categorical “normal” vs. “extreme” values and compared profiles of disease causal variants with those of neutral variations in gene sets 1 and 2. Table 4 reports the list of significantly distinct sequence/structural features for each variant category based on Fisher's exact test. Seven significant characteristics of causal variants found in the 57 genes in set 1 are: having a high deleterious count (≥4 out of 6 scores), introducing an amino acid change with large physico-chemical dissimilarity, inducing glycine or proline change, being situated in a protein domain, buried site or on a conserved protein surface, and causing at least mild protein destabilization. By contrast, negative control SNPs of this gene set were found to be enriched in conserved variants (conservation count = 3 out of 3) and generally locate in conformationally flexible sites.

These findings were validated with a parallel analysis of gene set 2, although in this case we gained statistical power by combining the set of epilepsy case variants with the non-epilepsy positive control SNPs (combined disease-SNPs *n* = 289). Each of the significant features detected in gene set 1 replicates in set 2, and additional evidences that disruption of disulfide bonds and location in conformationally rigid sites differentiate neutral and disease variants were obtained. These findings emphasize that causal variants are likely to affect protein functional sites, a conclusion that can only be obtained from structure-based analysis.

### Structural features predict deleteriousness of case SNPs

Next, we asked how the distribution of scores for the putative epilepsy-case variants compares with the negative and positive controls. By most of the standard deleteriousness scores, the distributions of cases are closer to the negative than the positive controls in both datasets (Figure 2). We conclude that there is little evidence from standard measures for enriched deleteriousness in the case variants from the epilepsy study. Similar observations were found for Grantham score, protein flexibility parameters, and a few other structural measures (data not shown).

However, we determined that the solvent accessibility measure (RSA) and a protein stability measure (ΔΔG) assign case variants to be more comparable to positive than negative controls (Figure 3A). Likewise, the same two structural parameters place “known epilepsy” variants in gene set 2 (Figure 3B) closer to the distribution observed in other disease-causing mutations. This analysis emphasizes the potential for structure-based deleteriousness measures to generate predictions that are more discriminating than those derived from measures of sequence conservation.

To obtain a list of high-priority functional nsSNPs for epilepsy, we applied the deleterious structure predictions enriched in positive SNPs (Table 4) to all candidate epilepsy variants and identified the ones with high SDSs (score ≥4 out of 7). A list of 14 high-likelihood structure-disrupted variants from 30 missense mutations was generated. To account for differences in the burden of mutations among genes, we used the Residual Variation Intolerance Score (RVIS) (Petrovski et al., 2013) to identify and compare the levels of mutational intolerance of each gene in our two datasets. The parameter determines the deviation of observed vs. expected numbers of common variants in a gene. Petrovski et al. (2013) found that genes which carry many common mutations (large positive RVISs) are less likely to influence disease development. Comparison of average RVIS between genes in sets 1 and 2 indicated that the two groups do not have the same tolerance to variations (*p*-value 0.0011, two-tailed *t*-test). The average RVIS for gene set 1 is 0.39 (range 0.11–0.67, 95% CI) whereas the value for gene set 2 is −0.40 (range −0.77 to −0.03, 95% CI). As expected, lower RVISs were observed for the documented disease causal genes (gene set 2). The finding is consistent with low RVISs in many OMIM genes (Petrovski et al., 2013). Nonetheless, the positive average RVIS for gene set 1 is not surprising; among the 57 genes in set 1, 38 genes (67%) are classified as being high tolerance.

**Figure 4:**
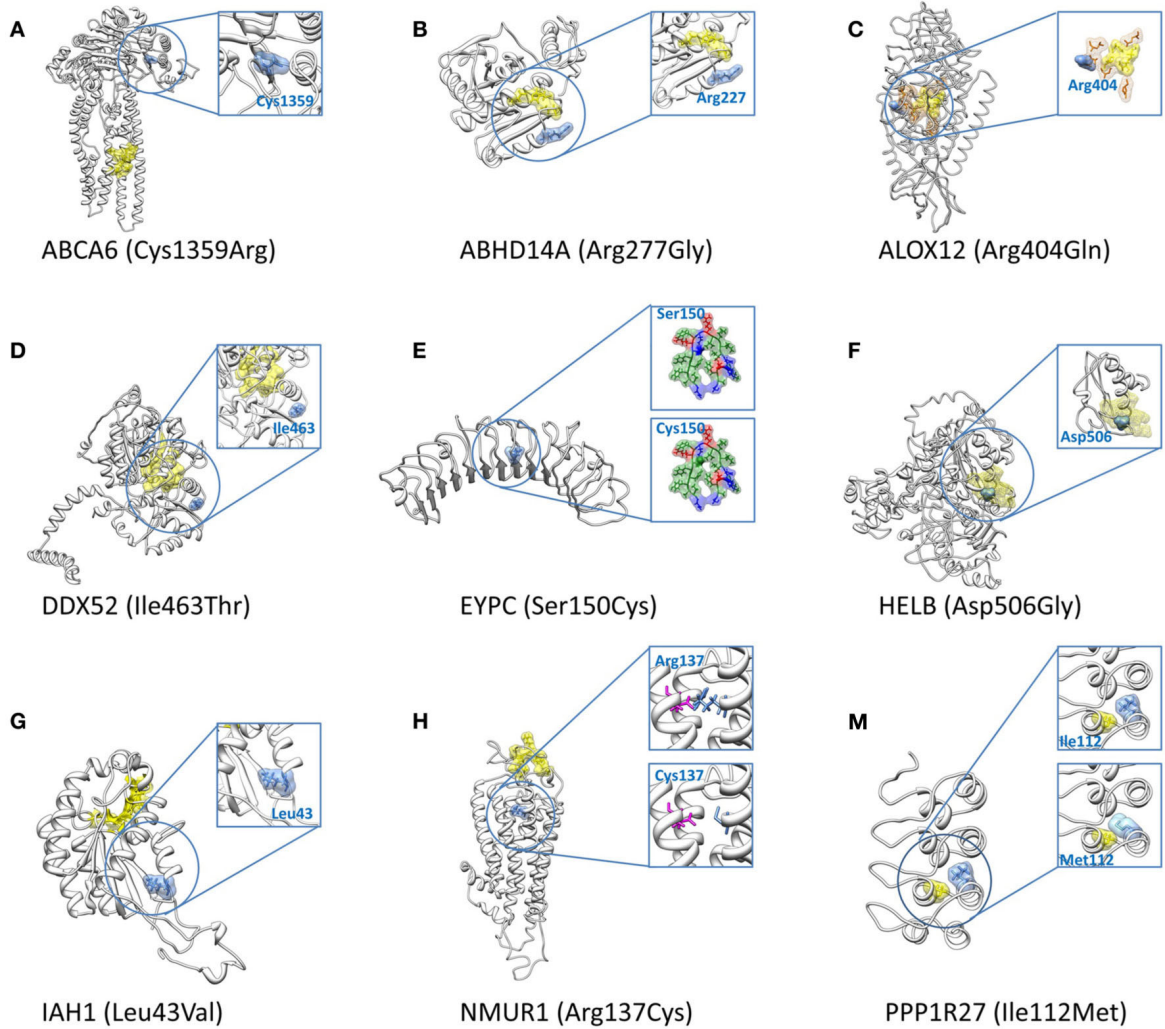
**3D structures for the nine high-priority variants for epilepsy**. The nine high-priority variants for epilepsy include eight structural disrupted variants **(A–H)** which locate in genes with high tolerance of polymorphisms (Table 5) and one structural disrupted variant **(M)** which resides in a low tolerance gene (Table 5). In all figures, mutated protein residues caused by the SNPs are indicated with blue surface representations along with the amino acid name (wild type) and residue index. Yellow surface representations refer to predicted binding sites or catalytic sites, except in **(M)** in which it represents a protein residue with close proximity to the altered amino acid. Orange representations in **(C)** indicate the predicted best conserved residue cluster on the protein surface. The substitution of Ser150Cys in **(E)** adds one favorable hydrophobic residue (green surface representation) to the core of the α/β horseshoe. The magenta sticks in **(H)** represent the partner residue, Glu117, which forms a salt bridge with wild type Arg137. This salt bridge is lost in the presence of mutant Cys at position 137.

Sub-classification of our 14 high SDS structure-disrupted variants yields 9 and 5 genes that are highly acceptable or tolerant of mutations, respectively (Table 5). Although variants in genes with low tolerance (negative RVISs) are more likely to be deleterious, our SDS focuses at the variant level, and the structural analysis potentially provides novel intuition that is not apparent from any sequence- or gene-based analysis. Therefore, the indication of many “high tolerance genes” in our dataset does not preclude potential functional effects of specific variants. For this reason, we performed in-depth literature searches on all 9 and 5 variants of the 2 subgroups, and provide our inference of the likelihood that a particular SNP may contribute to the epilepsy disorders (Table 6). Interestingly, we found that half (4 out of 9) of the SNPs in high tolerance genes have potential to promote epilepsy. The proportion is comparable (2 out of 5) for variants located in the low tolerance genes. Of the 9 variants in high tolerance genes, one has a structural feature that is compatible with those of neutral SNPs, i.e., the variant alters a highly flexible protein site; therefore, we disregarded it as a putative functional variant. Similar consideration of the low tolerance genes suggests that just one, *PPP1R27*, is likely to harbor a mutation that promotes epilepsy. This leads to prioritization of 9 “high-priority putative functional variants for epilepsy.” The locations of each of these variants with respect to their protein 3D structures are shown in Figure 4, and each is discussed below (variant numbering follows Table 5).

**Table 5:** **Case SNPs with high structural disruption scores**.

**Variant category**	**List**	**Gene**	**Variant position [base change, (amino acid change)]**	Structural disruption features^*^							**Structural disruption scores (SDS) (max = 7)**	**Additional gene/SNP features**	
				**High deleterious count? [Del count, max = 6]**	**Large amino acid change? [Grantham score]**	**Induce Gly/Pro change? [amino acid change]**	**Locate in buried site? [%RSA]**	**Locate on protein patch?**	**Locate in protein domain? [CATH architecture]**	**Reduce protein stability? [ΔΔG (kcal/mol)]**		**Gene tolerance level [RVIS]**	**Locate in highly flexible site [percentile of P(flexible)]**
Structural disrupted variants which locate in high tolerance genes	A	*ABCA6*	(4075)TGC > CGC [C1359R]	Yes [6]	Yes [180]		Yes [2.05]		Yes [3-layer(αβα) sandwich]	Yes [1.64]	5	high [0.26]	
	B	*ABHD14A*	(685)CGA > GGA [R227G]		Yes [125]	Yes [R > G]			Yes [3-layer(αβα) sandwich]	Yes [0.87]	4	high [0.77]	
	C	*ALOX12*	(1211)CGG > CAG [R404Q]	Yes [4]			Yes [10.55]	Yes	Yes [up-down bundle]	Yes [0.90]	5	High [0.8]	
	D	*DDX52*	(1064)ATC > ACC [I463T]	Yes [4]			Yes [4.42]		Yes [3-layer(αβα) sandwich]	Yes [1.37]	4	High [0.05]	
	E	*EPYC*	(449)TCC > TGC [S150C]	Yes [5]	Yes [112]		Yes [0.77]		Yes [α−β horseshoe]		4	High [0.51]	
	F	*HELB*	(1517)GAT > GGT [D506G]	Yes [4]		Yes [D > G]	Yes [18.27]			Yes [1.40]	4	High [1.08]	
	G	*IAH1*	(127)CTG > GTG [L43V]	Yes [4]			Yes [4.66]		Yes [3-layer(αβα) sandwich]	Yes [2.06]	4	High [0.17]	
	H	*NMUR1*	(409)CGC > TGC [R137C]	Yes [4]	Yes [180]		Yes [1.17]		Yes [up-down bundle]	Yes [1.83]	5	High [0.27]	
	I	*PALB2*	(2993)GGA > GAA [G998E]	Yes [5]		Yes [G > E]	Yes [2.27]			Yes [3.23]	4	High [0.32]	Yes [97.75]
Structural disrupted variants which locate in low tolerance genes	J	*EXOG*	(830)GGA > GTA [G277V]	Yes [6]	Yes [109]	Yes [G > V]			Yes [3-layer(αβα) sandwich]	Yes [1.64]	5	Low [−0.45]	
	K	*FAAH2*	(821)CGT > CAT [R274H]	Yes [4]			Yes [0]		Yes [α−β complex]	Yes [0.95]	4	Low [−0.29]	
	L	*MAOA*	(374)AAT > AGT [N125S]	Yes [4]			Yes [6.65]		Yes [orthogonal bundle]	Yes [1.36]	4	Low [−0.14]	
	M	*PPP1R27*	(336)ATA > ATG [I112M]	Yes [5]			Yes [0]		Yes [α horseshoe]	Yes [1.41]	4	Low [−0.32]	
	N	*PTPN14*	(566)GAA > GGA [E189G]	Yes [4]		Yes [E > G]			Yes [up-down bundle]	Yes [1.79]	4	Low [−0.30]	

*Each candidate epilepsy variant is suggested to disrupt protein structure/function if its structural disruptions score (SDS) is high (≥4 out of 7). A list of 14 putative structural disrupted variants from 30 missense mutations is reported, along with the corresponding 7 scores that make up the SDS. The variants are classified into two groups, based on the level of polymorphism tolerance of its gene*.

* *Only the values corresponding to enriched characteristics of causal SNPs are included in the table; the empty cells do have values but they are not presented here for clarity*.

**Table 6:** **Summary of structural disrupted case SNPs**.

**Variant category**	**List**	**Gene**	**Variant position [base change, (amino acid change)]**	**Gene functions [biological function]**	Variant's features^*^	**Disease implications^†^**	**Epilepsy implications^‡^**	**Structural disruption scores (max = 7)**	**% MAF (AfrAmr, EurAmr)**
Structural disrupted variants which locate in high tolerance gene	A	*ABCA6*	(4075)TGC > CGC [C1359R]	ABC transporter A family member 6 [lipid homeostasis]	Large change in amino acid properties, mutation causes protein destabilization but does not alter disulfide bonds	n/a	n/a	5	0.30, 2.00
	B	*ABHD14A*	(685)CGA > GGA [R227G]	Hydrolase [neuron development]	Near active site (low Γ), loss of side chain (R > G)	Linked to Chanarin-Dorfman syndrome (fat depositions in internal organs)	Less likely	4	0.16, 0.23
	C	*ALOX12*	(1211)CGG > CAG [R404Q]	Lipoxygenase [lipid metabolism]	Near active site, on best protein patch	Shares substrate with COX (COX-2 expression increases upon electrical stimuli)	Likely	5	0.05, 0.37
	D	*DDX52*	(1064)ATC > ACC [I463T]	RNA helicase [mRNA degradation control]	Stabilization center	Gain of function in Drosophila's homolog suppresses seizure; mRNA loss accounts for 1/3 of human diseases	Maybe	4	0.23, 1.16
	E	*EPYC*	(449)TCC>TGC [S150C]	Epiphycan [cartilage development]	Stabilization center, mutation yields preferred hydrophobic core	Osteoarthritis	No	4	0.61, 2.42
	F	*HELB*	(1517)GAT > GGT [D506G]	DNA helicase [DNA damage repair]	3 indications as a binding residue, confirmed by mutagenesis	Effective cellular protection mechanism helps animals survive brain injuries after induced seizures	Likely	4	0.57, 3.76
	G	*IAH1*	(127)CTG > GTG [L43V]	Esterase [lipid metabolism]	Mutation locates at a turn region (not favorable in highly structured proteins)	Antiepileptic drugs interfere with lipid metabolisms	Likely (drug response)	4	0.28, 2.62
	H	*NMUR1*	(409)CGC > TGC [R137C]	Neuromedin-U receptor 1 [uterus contraction, vasoconstriction]	Diminishes stabilizing salt bridge and causes protein destabilization	Control of food intake	No	5	0, 0.08
	I	*PALB2*	(2993)GGA > GAA [G998E]	Partner and localizer of BRCA2 [homologous recombination repair]	Mutation may interfere with conformational flexibility of protein, largely decreases protein stability	Several mutations identified in breast cancer but disease associations are not definitive	No	4	0.59, 2.40
Structural disrupted variants which locate in low tolerance gene	J	*EXOG*	(830)GGA > GTA [G277V]	Mitochondria endonuclease [programmed cell death]	Rigid residue at turn region, controls positioning of C-terminal and active site, confirmed by mutagenesis	n/a but reduces substrate binding	n/a	5	0.27, 1.20
	K	*FAAH2*	(821)CGT > CAT [R274H]	Fatty-acid amide hydrolase2 [lipid metabolism]	Mildly decreases protein stability	Gene's regulation of endocannabinoid system is linked to Alzheimer's and other CNS disorders	Maybe	4	n/a
	L	*MAOA*	(374)AAT > AGT [N125S]	Monoamine oxidase type A [neurotransmitter metabolism]	Far from functional sites, mildly reduces protein stability	Gene catalyzes several neurotransmitters and associated with behavioral phenotypes, confirmed by animal studies	No	4	n/a
	M	*PPP1R27*	(336)ATA > ATG [I112M]	Phosphatase regulator [cellular process regulation]	Longer amino acid side chain causes steric clash	Member of KEGG epilepsy pathway; protein in the same family linked to Lafora disease (teenager-onset of epilepsy)	Likely	4	n/a
	N	*PTPN14*	(566)GAA > GGA [E189G]	Tyrosine-protein phosphatase non-receptor type 14 [cellular process regulation]	Mutation does not alter inter-residue bonding but slightly decreases protein stability	Several mutations identified in colorectal cancers	No	4	0.93, 3.22

*The table summarizes the structural and clinical findings for each of the top 14 case variants. The 9 putative functional variants for epilepsy (variants A–H, M) are identified from of a subset of the 14 variants. Eight variants (variants A–H) are located in “high tolerance genes” and do not possess a compatible feature with those of neutral SNPs. The ninth variant (variant M) is located in a “low tolerance gene”; the gene is likely to be associated with epilepsy disorders*.

* *Variant's features include all structural changes/implications that were collected during the analysis, regardless of their significant in feature enrichment toward causal SNPs*.

† Disease implications denote any clinically-relevant associations found in literatures.

‡ *Epilepsy implications indicate our opinions on whether or not the variant contributes to epilepsy development. The opinion is based upon several data sources. However, the considerations exclude the SDS of a variant and its minor allele frequencies (MAFs). (The SDS had already been utilized as a filter during the variant prioritization step. The allele frequencies are presented here solely for comparison purposes)*.

### Individual assessment of putative structurally deleterious variants

#### Cys1359Arg in ABCA6

Cys1359 mutation is located in a buried site of ABCA6, a protein that plays roles in macrophage lipid homeostasis (Figure 4A). Despite the high SDS of this variant (score 5/7), it has not been associated with diseases. The functionality of cysteine residue depends largely on the protein structure and its cellular location. For this instance of Cys to Arg change, it does not alter the pattern of disulfide bonds, partly due to the rarity of this bonding type within membrane proteins (Betts and Russell, 2003). However, the C1359R mutation is still considered as a crucial change (Grantham score 180, ΔΔG 1.64 kcal/mol), especially when the mutation occurs in the middle of protein domain.

#### Arg277Gly in ABHD14A

Arg277 is located on an exposed site of ABHD14A (Figure 4B). Another member of this protein family, ABHD5, is thought to be responsible for a rare genetic disorder called Chanarin-Dorfman syndrome. This is the only variant among the 14 structural disrupted case SNPs that was not detected as “deleterious” by any sequence-based algorithms (deleterious count 0/6), but our SDS suggests it has potential impact on the protein (SDS 4/7). The wild type Arg227 residue has low sequence optimality (Γ −0.58 kcal/mol), a likely indicator that it has close proximity to a catalytic site. Moreover, the Arg to Gly change is considered as an unfavorable substitution, especially when it occurs in a structured protein, as the lack of a side chain in Gly may diminish proper protein folding or intermolecular interactions.

#### Arg404Gln in ALOX12

Arg404 is found in a buried site of ALOX12 (Figure 4C). Arg404 is in close proximity with the catalytic site of this protein and is also predicted to be part of the protein patch for intermolecular interactions. The Arg404Gln substitution within this protein is also predicted to cause a slight protein destabilization (ΔΔG 0.90 kcal/mol), despite the acceptable Grantham substitution score (<100). We suspect the variant may play roles in epilepsy implication, since a link between its substrate (arachidonic acid) and seizure susceptibility had been proposed (Cole-Edwards and Bazan, 2005).

#### Ile463Thr in DDX52

Ile463 is located in a buried site of DDX52 (Figure 4D). A study of seizure susceptibility in *Drosophila* discovered that a gain-of-function mutation in the *maleless* helicase gene can suppress seizure susceptibility in bang-sensitive *Drosophila* mutants (Kuebler et al., 2001). The Ile463Thr mutation in this protein affects the structural integrity of the protein since it is detected as a SC, consistent with its destabilization effect (ΔΔG 1.37 kcal/mol).

#### Ser150Cys in EYPC

The Ser150Cys variant is located in the core of the leucine-rich repeat (LRR) structural motifs of EYPC (Figure 4E). Several disulfide bonds are formed between cysteine clusters that flank the LRRs, providing additional structural support, but no changes of disulfide bonds or hydrogen bonds appear in the mutated protein. Ser150Cys has minimal impact on protein stability (ΔΔG = −0.41 kcal/mol), and while the substitution induces a large physico-chemical change (Grantham score ≥ 100), the substitution is considered neutral if located in α/β protein (Xu et al., 2010). Indeed, we observed Ser150Cys added one favorable hydrophobic residue to the core of the α/β horseshoe. Despite its high SDS (4/7), we consider it unlikely that this variant contributes to any disorders.

#### Asp506Gly in HELB

Asp506 is situated in a buried site of HELB (Figure 4F) and several of its features are predicted to interfere with ligand binding. First, wild type Asp506 has an exceptionally low sequence optimality value (Γ −4.14 kcal/mol). Second, wild type Asp506 is predicted to be a highly flexible site by three parameters, although the values are not extreme. Third, the Asp506Gly substitution is predicted to destabilize the protein (ΔΔG 1.40 kcal/mol). These characteristics coincide with a recent mutagenesis experiment that proves Asp506 is part of a binding motif, and the mutation of D506A induces loss of substrate binding when associated with E499A and D510A (Guler et al., 2012). Study of another human DNA helicase, Twinkle, demonstrated that two missense mutations were detected in patients with a wide range of psychiatric symptoms, including severe epileptic encephalopathy, possibly due to inefficient recovery from molecular stress (Lonnqvist et al., 2009). HELB Asp506 is thus particularly interesting for further assessment of a role in epilepsy.

#### Leu43Val in IAH1

Leu43 is located at a buried site in IAH1, although it lies far from the substrate binding site (Figure 4G). The protein is of interest given that many antiepileptic drugs are potent enzyme inducers and inhibitors of the cytochrome P450 system, which affects lipid and glucose metabolisms, as well as evidence that increased lipase level is one of the side effects of anti-psychotic and anti-epileptic drugs (Voudris et al., 2004, 2006). Substitution of Leu to Val is quite favorable in all protein folding types (Xu et al., 2010), but Leu43Val in this protein is suspected to reduce protein stability (ΔΔG 2.06 kcal/mol). A plausible explanation may be that this protein is highly structured, comprising only a few loop residues. The turn regions may play an essential role in bringing together and enabling or allowing interactions between regular secondary structure elements.

#### Arg137Cys in NMUR1

Arg137 is situated in the central cavity of NMUR1 (Figure 4H). The presence of the a mutant Cys at position 137 diminishes the stabilizing salt bridge between wild type Arg137 and Glu117, and results in a decrease in protein stability (ΔΔG 1.83 kcal/mol). The gene has no known associations with any diseases.

#### Ile112Met in PPP1R27

Ile112 is found at a buried site of PPP1R27 (Figure 4M). A study of a similar protein, PPP1R3C, identified one missense mutation that may lead to a mild phenotype in Lafora disease—a teenage onset epilepsy disorder (Guerrero et al., 2011). In addition, protein phosphatase 1 (PP1) was identified as a member of long term potentiation (PTP) pathway in epileptogenesis and epilepsy (KEGG: map04720) (Kanehisa and Goto, 2000; Lukasiuk and Pitkanen, 2012). Other genes in this pathway mostly regulate neurotransmission and ion channel receptors. The Ile to Met substitution is quite favorable both in general (Grantham score < 100) and in distinct types of protein folding (Xu et al., 2010). However, the longer aliphatic side chain of Met creates steric clashes with Ala104 of an adjacent helical region. This single point mutation is predicted to destabilize the protein (ΔΔG 1.41 kcal/mol).

### Structural disruption score correlates with sequence-based deleterious score

Finally, we tested whether the SDS correlates with measures not used to construct the score itself. We performed an additional sequence-based deleterious prediction of case SNPs using Condel: a weighted score that integrates the output of five tools (Gonzalez-Perez and Lopez-Bigas, 2011), three of which were used in our analysis. There is a significant positive correlation between our SDS and the Condel score (*p*-value 0.0342, *n* = 30). The trend persists after removing the sequence-based deleterious scores from SDSs of 16 variants (the variants have deleterious count ≥ 4 out of 6), although the significance in correlation is reduced to a marginal level (*p*-value 0.0717). In addition, we performed a similar analysis by sequentially removing one SDS component from the total score, and found the significantly positive correlation between SDS and Condel is maintained (Table 7). The weakest structure-based parameter among all of the SDS components is the classification of buried vs. exposed site using RSA. After removing this indicator from 15 variants, we did not detect any correlation between the two measures (*p*-value 0.13). Note that this analysis is complicated by the small sample sizes. Nonetheless, the finding supports our expectation that although the deleterious count is one of the major components for constructing our SDS, the remaining non-conventional deleteriousness parameters also have substantial impact on the overall missense variant evaluation.

**Table 7:** **Step-wise analysis for correlation of SDS and Condel score**.

**SDS parameters**	***R*^**2**^ of linear fit**	***P*-value of correlation**	**# of variants affected by the revised SDS^†^**
All SDS	0.1504	0.0342^**^	none
SDS-high deleterious count	0.1112	0.0717^*^	16
SDS-large amino acid change	0.1508	0.0340^**^	5
SDS-induce gly/Pro change	0.1308	0.0496^**^	7
SDS-locate in buried site	0.0782	NS (*p* = 0.1344)	15
SDS-locate on protein patch	0.1629	0.0270^**^	3
SDS-locate in protein domain	0.2093	0.0110^**^	25
SDS-reduce protein stability	0.1243	0.0560^*^	20

*Initial SDS includes 7 parameters, described in Table 4; the maximum SDS for each variant is 7. The revised SDS calculates the score by exclude one SDS component at a time; the maximum revised score for a variant equals 6*.

† The full dataset has 30 missense variants. All data points were used to test for a correlation between SDS and Condel score. When an SDS component was removed during the step-wise analysis, the SDSs for some numbers of variants were affected, i.e., the excluded parameter was applicable to the variant. For such cases, the correlation analysis was performed with all of the 30 data points, minus the number of exclusions indicated in the last column. Significant p-values are designated with “

**” and “*” *for α = 0.05 and α = 0.10, respectively. Non-significant test statistics are labeled with NS, followed by the correspondent p-value*.

## Discussion

### Current perspectives in prioritization of epilepsy variants

The evaluation and prioritization of candidate case variants is particularly difficult when the disorder involves a dissimilar set of genes, as is the case for epilepsy disorders, which are now known to involve diverse molecular pathways (Noebels, 2003; Garofalo et al., 2012). The classic epilepsy genes (ion channel genes/regulatory genes, neurotransmitter genes/receptor genes/regulatory genes, genes that disrupt cortical circuits, and genes that lower the convulsion stimuli) rarely present in genomic data from sequencing studies. This may be because the classical epilepsy mutations are Mendelian, whereas exome sequencing likely targets more polygenic cases, noting that only 1% of epilepsy disorders are inherited in a Mendelian manner (Cavalleri and Delanty, 2012).

Ferraro et al. (2012) highlighted how daunting the task is for epilepsy when they exemplified some factors in the design of cohort studies that influences the discovery of true positive epileptic variants: the selection of cases (presence/absence of the cause of symptoms), the seizure types (determine the amount of genetic influences), the patient profiles (age at onset, gender, characters of seizure incidences, etc.), and the assumption of genetic patterns (common variant effects, rare variant effects, or a combination of both) (Ferraro et al., 2012). Some authors are starting to incorporate disease information as prior knowledge in the probabilistic evaluation of candidate causal SNPs (Yandell et al., 2011; Sifrim et al., 2013; Robinson et al., 2014), but the scarcity of knowledge related to epilepsy genes limits this approach. Indeed, we found that the genes in our dataset are somewhat poorly understood and their disease contributions are largely unknown.

In addition to SNPs, structural variations have been shown to associate with epilepsy genetics. Jia et al. (2011) used stepwise enrichment analysis of protein-protein interactions to derive a molecular network of 20 high priority candidate genes linked to copy number variation (Jia et al., 2011). Interestingly, the genes do not overlay with the 68 homozygous nsSNP-containing genes in our dataset [but they do overlap with 4/1604 genes that harbor heterozygous variants: (Heinzen et al., 2012)]. An independent comparison with nine genes that harbor *de novo* mutations (identified from trios—unaffected parents and their affected child) also found no overlap (Epi4k Consortium and Epilepsy Phenomegenome Project, 2013).

Consequently, we cannot be sure that any of the variants discussed in this article are truly causal for epilepsy. However, the variant prioritization scheme does suggest a reduced number of candidates which, on the basis of careful curation of protein structure, might be taken forward for targeted experimental manipulation and assessment of biological function in cell lines or model organisms.

### Key findings

We have developed a structure-based variant analysis protocol that evaluates the effects of missense mutations with respect to their predicted effects on protein features, such as solvent accessibility, stability, and flexibility. Replicated trends for putative case SNPs to have aberrant structural features that more closely match those of established disease mutations in the same proteins, than to those of neutral polymorphisms, establish the potential utility of this approach as an orthogonal protocol to sequence-based assessment of deleteriousness.

Starting with 71 genes harboring putative case SNPs from an exome sequencing study of epilepsy disorders (Heinzen et al., 2012), we were able to perform the assessments on 57 gene products. Presumably only a fraction of these are actually causal, so our expectation was simply that the distribution of risk scores may be shifted from neutral toward disease-associated. Several features were observed to classify SNPs into two groups: putative functional variants, or presumably neutral variants, and a composite risk score based on summation of these features highlighted nine putative functional variants, from thirty exclusive missense mutations whose protein 3D structures are available. Although none of these has been previously linked to epilepsy disorders, detailed case-by-case analysis strongly suggests that several should be prioritized for further functional evaluation.

Although our structure-based analysis only captured a fraction of variant residues due to the limited availability of 3D structures (Table 1), we show that 44, 32, and 75%, of case, negative, and positive variants from the first gene set are amenable to structure-based predictions. The equivalent percentages for set 2 genes are 49, 37, and 51%, respectively. More importantly, we were able to represent 84% of the proteins in our first set, and 86% in the second set, despite the difficulty in generating high quality structures. Our preliminary analyses suggest that it is not necessary to have structural data for all variants in order to construct the SDS. Since the sequence-based predictions of variants with structure data are similar to those obtained for all SNPs in the dataset (data not shown), we are confident that the conclusions from our structure-based variant assessment protocol can be extended to the complete SNP pools in each of the two gene sets.

With our combined sequence- and structure-based analysis pipeline, we discovered that some features are predominantly found among negative or positive SNPs. Structure-based parameters contribute as much as 50% of the features that differentiate the two types of variants. There are several key observations from our feature enrichment analysis. First, we noticed seven common characteristics that are predominantly found in the positive control SNPs, regardless of the set of genes. SNPs with strong effects are those that: have deleterious count ≥ 4, have Grantham score ≥ 100, induce glycine or proline changes, locate in protein domains, in buried sites, or on conserved protein patches, and destabilize the protein. Second, variants with neutral effects (negative SNPs) have a few strong enriched features. In gene set 1, we found negative SNPs are more likely to be in conformationally flexible sites. A similar feature was detected in gene set 2, in which non-damaging SNPs are mostly highly dynamic sites. An additional distinctive characteristic of the negative SNPs in gene set 1 is that they tend to affect highly conserved residues. This finding appears to be counter intuitive; we suspected that this unique observation seems to be an exception for this particular gene group.

### Study limitations

A primary limitation of our approach is the requirement for homology models that support computational prediction of structural characteristics. Specifically, only 18/68 proteins had at least partial experimental structures, so homologous templates were used in most cases. These were not available for just 9% of the proteins (*n* = 6), but the retained models did not cover the disrupted site for another 56% (36/68 variant sites), and 24 of the potentially disrupted proteins are larger than 1000 amino acids, which also presents additional challenges for building models and satisfying quality settings. Nevertheless, by restricting the modeling to domains, we were able to model 84% of the 68 candidate genes (covering 44, 32, and 75%, of case, negative, and positive variants). This is a clear improvement on the automated pipelines used in training algorithms such as PolyPhen2. We also ensured that the quality of the models was validated wherever possible, which also introduces an intensive manual curation requirement into the analytical pipeline, requiring some knowledge with methods that most genomicists are not familiar with.

An analytical limitation is that the size of the dataset is relatively small, since only one variant per gene was studied, and just 68 proteins were available to begin with. Since these are structurally diverse, it is likely that different aspects of protein structure are affected and the probability of enrichment for any one structural feature is correspondingly reduced. While approximately three quarters of amino acid changes leading to Mendelian diseases consistently induce protein destabilization (Yue et al., 2014), the structural consequences of missense variants in complex diseases such as the epilepsy disorders are likely to be of a more diverse nature.

### Study innovations

The SDS offers a novel strategy for genomic profiling of variants that have uncertain but likely weakly deleterious function. It combines similar instances of variants with respect to their predicted impact on aspects of protein structure, allowing joint assessment of the impact of the variants as a class on biological function. Instead of evaluating each variant one by one, SDS provides a ranking that might be used to guide downstream experimental and/or clinical evaluations.

Other studies using structural biology approaches to examine the variant effects have considered a large number of variants per gene, facilitating direct contrasts of variant characteristics and predictions for individual genes (Dybowski et al., 2011) or genes with similar structures/functions (Jordan et al., 2011; Izarzugaza et al., 2013). The genomic data that we started with is relatively small by comparison. However, we provide an alternative structure-based approach that can accommodate the small number of variants expected from exome sequencing samples as well as the large diversity of gene functions they will typically generate.

Most importantly, our SDS score implementation assures that case variants share similar characteristics as those observed in causal variants, but not neutral variants known to reside in the same proteins. We have validated the findings in a replication dataset, and show how the approach can be used as a unique solution to prioritizing case variants in unrelated genes. Though not providing a guarantee of disrupted function, the score should be considered as a complementary approach to existing sequence-based deleteriousness prediction.

### Conclusion

Using the list of enriched features, we concluded that this novel structure-based assessment protocol for missense variant deleteriousness has a potential to determine high-priority candidate variants suitable for experimental validations. The analysis may prove to be useful, particularly when traditional sequence-based predictions are inconclusive. An important question is whether the same structural attributes differentiate neutral and functional variants for different categories of diseases.

Because our study employed large numbers of external resources (variant predictions, gene information, 3D structure modeling and quality controls, and sequence- and structure-based predictions), the analysis pipeline presented here is not readily automated. Aspects of it are in theory readily generalizable to all classes of proteins, and once all the above steps have been accomplished, the variant deleteriousness structure-based predictions could be effectively populated into a database. After that labor-intensive step is completed, the SDS for any variants in a dataset may be computed and retrieved virtually by combining the predictions for the genes specific for the study.

## Author contributions

Thanawadee Preeprem carried out the analysis. Thanawadee Preeprem and Greg Gibson participated in the design of the study and wrote the manuscript. Both authors read and approved the final manuscript.

### Conflict of interest statement

The authors declare that the research was conducted in the absence of any commercial or financial relationships that could be construed as a potential conflict of interest.
